# Cases from Dr. Hamilton’s Clinic at the Buffalo Hospital of the Sisters of Charity

**Published:** 1859-03

**Authors:** J. Boardman


### BUFFALO MEDICAL JOURNAL

AND

### MONTHLY REVIEW.

**VOL. 14.** MARCH, 1859. **NO. 10.**

### ORIGINAL COMMUNICATIONS.

ART. I.—*Cases from Dr. Hamilton's Clinic at the Buffalo Hospital of the Sisters of Charity.* Reported by J. BOARDMAN, M. D.

*Nævi.* ——, aged three years, was brought before the class, with a nævus, or mother's mark, upon the right hand. It was found upon the child at birth, and had not extended or changed its appearance, till within a few weeks, at which time the mother discovered a small ulcer upon it, which was slowly increasing in size, notwithstanding all of her efforts to heal it. It was this ulcer that induced the mother to bring the child to Dr. Hamilton. Upon examination of the right hand, there was found a large red, or purple discoloration of the skin, very slightly elevated above the surface, extending from the wrist, over the back of the fingers, and nearly surrounding the base of the thumb and the base of the fore-finger. There was no pulsation; it had been without pain, and the abnormal structure was confined to the skin itself, not involving any of the tissues underneath. A small, superficial, sloughing ulcer, about half an inch in its longest diameter, was to be seen upon the nævus, between the thumb and fore-finger. This was the only mark of the kind upon the child.

Dr. Hamilton remarked, that in this case, nature was undertaking the cure of the disease, and that he should not seek to cure the ulcer at present. He thought, probably, the ulcer would not extend over the whole of the diseased surface. Inflammation and ulceration were often artificially produced by the surgeon, to cure nævi, and, as nature had taken upon herself this duty, it was best not to interfere. If he should be able to cure the ulcer at present, the probability was, that the hand would be the source of much trouble, for with such proneness to ulceration, each scratch, abrasion of the skin, or even contusion of the surface, would, very likely, be followed by ulceration, which would be a source of great trouble. A gently stimulating ointment was ordered for the child.

A., aged 13 years, was brought before the class, with a nævus upon the face. An examination showed an oval tumor, extending from a point below the right eye, a little above the alæ of the nose, diagonally downward and backward, toward the point where the facial artery passes over the inferior maxilla. The diameters were about one and three-quarter inches by one inch, and it was elevated already three-eighths of an inch above the level of the skin. The tumor was of a brownish purple hue, and upon its surface were to be seen a few rather coarse hairs. It was without pulsation, and never had been the seat of pain, save that in winter it was liable to crack, bleed, and slightly ulcerate. It was congenital, but his father thought it had increased a little in size of late.

Dr. Hamilton advised the removal of the tumor, not only because of the tendency to increase, but because this form was prone to degenerate in time into a malignant growth. He called the attention of the class to the principal difficulty of this case — the removal and consequent loss of so much skin from the side of the face. Nature heals a wound in which there has been a loss of the integuments in two ways: by the production of new skin and, also, by a contraction, or pursing in, of the surrounding skin, as can be seen in the healing of burns, or of ulcers upon the leg. Where the parts are comparatively free, the contraction will be the greatest. In the present case, there might be danger of ectropion of the lower lid being produced, or of the angle of the mouth being lifted and drawn backwards. If this tumor were destroyed by the ligature, or by the caustic, he thought the scar would be more irregular, and the danger of deformity much greater, than if the tumor was removed by the knife. Many times, when the surgeon removes such a quantity of skin, that deformity, from contraction, which is liable to ensue, he can, in part, or entirely, overcome, by either transplanting a piece of skin from some other part, or by sliding some of the adjacent skin towards the wound. Here he could not obtain a supply either from the temple or forehead. The distance was too great, for always there must be a sufficient pedicle left to support the lip of the flap, until it becomes firmly attached to the parts to which it has been transplanted. He was unwilling to turn a flap *upwards* from the neck, for his experience had taught him that, in these flaps turned upwards, sloughing was very apt to occur, sometimes destroying almost the entire flap, even in those instances where a thick flap could be obtained, but a neck flap must necessarily be rather thin. He could not extend the incisions towards the ear, and slide forward a piece of skin, with any hope of success; for the ear would be a fixed point, and the alæ of the nose, and the angle of the mouth were movable, therefore they would yield, and deformity would ensue. In this case he should seek to make the upper part unite by means of sutures, in such a way, that the danger of ectropion would be very little, if any, leaving the lower portion of the wound open, he hoped new skin would be formed, so that the drawing of the angle of the mouth would be slight. He then proceeded to operate, drawing the lines of his incision close around the base of the tumor, with tincture of iodine, as is his custom in all plastic operations, and in those operations where he wishes to save all the integument possible. With his knife he then made an incision around the whole tumor, and dissected it carefully away. He had expected a copious hæmorrhage, but, although blood freely followed the knife, yet a moment's pressure with the sponge restrained it, and there was but one vessel that required the ligature. The upper edges of the wound were brought together with three interrupted sutures, the lower one of which Dr. Hamilton said, he expected would be torn ont, but, in the mean time, he expected the two other sutures would have caused the integument to unite. The wound was then dressed with simple cerate and lint.

Dr. Hamilton divides this disease into three principal classes:

1st. Capillary.
2d. Venous.
3d. Arterial.

The capillary form appears as slightly elevated spots or patches upon the skin, having a red, or purplish hue. It involves only the skin, and is an enlargement and multiplication of the capillary vessels of this tissue. It is always congenital, and though often, at birth, of an exceeding small size, it is not unusual to find it, in time, the cause of considerable deformity. The most frequent seat of this disease, Dr. Hamilton thinks, is upon the cheek, a little below the centre of the eye; and though nævi are most frequently found upon the head, neck, and arms, occasionally they are found in almost every part of the body; often appearing as but the enlargement of a few capillaries, which may remain without change throughout life, or without any known cause, suddenly they may take an increased action, and spread over more or less of the surrounding tissue; ulceration may ensue, and cause the destruc- of a part, or even the whole, of the nævus; for this tissue seems to have a much lower vitality than healthy skin. No pulsation is to be found in the tumor, but this, like the other varieties, if wounded, is exceedingly prone to hæmorrhage.

The second variety is found principally of enlarged veins, and may be situated entirely in the skin, or may involve the subcutaneous tissue. The arteries supplying the part are slightly enlarged, but constitute a small part of the tumor, compared with the veins. It is more elevated than the preceding variety, and has a soft, doughy, inelastic feel, and is without pulsation. It generally is of a bluish, or bluish-purple hue, though sometimes of a brownish cast. It does not always affect the skin, but may be confined to the tissue immediately underneath. When wounded, there is a copious flow of venous blood, but the hæmorrhage is generally controlled by moderate pressure. This is a much more dangerous form than the preceding, life being endangered not only by the frequent bleeding, but also by the proneness of the tumor to degenerate in character. Miller writes:

"Medullary or melanotic matter may be deposited in and around it, or come altogether to take its place — the original character of the growth being entirely lost. This I have seen occur in an erectile tumor of the cheek."

The third variety, or that form which may be known as aneurism by anastomosis, depends almost entirely upon the arterial enlargement and increase, for its formation, and unless assistance is rendered, is almost certain, in time, to terminate the life of the patient by the loss of blood. It is most frequently found upon the head, neck, and upper extremities. Originating in the tissues underneath the skin, it forms an elevated tumor, irregular in shape, soft, slightly elastic, and compressible, having a spongy feel. A pulsation, or thrill, is to be felt in the tumor. As the skin becomes involved, it assumes a reddish purple hue. The vessels leading into the tumor are much enlarged, and carry a plentiful supply of arterial blood to the tumor. The tissue is almost identical with normal erectile tissue, "but with this difference, that whereas in the normal, there are periods of complete repose and collapse, tension and fullness occurring, but occasionally by local determination; in the morbid, there is never utter placidity and repose. The tumor is more full and tense at one time than at another; yet at all times it is full and active, evincing an undulatory movement, if small; when large, it may be found pulsating strongly, and with *bruit*, as in ordinary aneurism.

In all cases, its bulk is temporarily increased by mental excitement, muscular exertion, and whatever suddenly and much excites the circulation."

In regard to the structure of nævi, comparatively little is known, especially concerning the connection of the different vessels with each other. Paget writes:

"As in the natural development of parts, so in what is morbid, organization, to a certain point, precedes vascularity, and the formation of blood-vessels follows on that of the growths into which they pass. But here the case appears reversed. The calibre of the blood-vessels increases, and the solid tissues between them diminish; all the growth of an erectile tumor is an enlargement of blood vessels, with diminution of the tissues in which they ramify; or, rather, it is often an enlargement, not of blood vessels, but of blood spaces: for though, in the first stages of the disease, the walls of the vessels may grow, and elongate, so that the vessels become tortuous, yet, after a time, the walls waste rather than grow; apertures seem to form through mutually apposed blood-vessels. Hence, at last, in place of branching and anastomosing tubes, there is only a network formed of the remains of their walls."

Nævi, or vascular tumors, are, by far, the most common of congenital tumors. The first two varieties, I believe, are always congenital, never originating after birth; but the third variety occurs at any period of life. It may originate from an injury, or without any apparent cause. I have seen an instance of this, originating from a slight blow upon the scalp, which resisted different attempts to effect a permanent cure, and eventually caused the death of the patient. I know that some writers speak of nævus, as a disease that may begin at any period of life, but I think this remark will apply only to this last variety. All varieties are prone to a rapid growth during the period of childhood; but they may remain inactive throughout a long life, never increasing beyond their first discovered size, or at any time, even after fifty years of inactivity, rapid growth may begin, or they may, of themselves, spontaneously disappear. At times, active growth takes place, resulting in ulceration, which terminates the life of the tumor. This fortune, however, is to but a few. In most cases where active growth has commenced, the patient must receive aid, or he will die from hæmorrhage, as it will certainly take place.

The surgeon endeavors to accomplish a permanent cure, in one of three ways. Either by producing a change of structure in the tumor, by arresting the circulation in the diseased mass; or, by the destruction, or remova of the diseased tissue.

*By producing a change of structure.* This may be at times accomplished by pressure. Seven or eight years since, a man came into this hospital, with a small vascular tumor situated on the roof of his mouth. Having a clay pipe in his month, a blow upon the bowl, caused the end to lacerate the mucous membrane of the roof of his mooth. From this, a vascular tumor, the size of a small bean grew, which, in ten days, the time of his first presenting himself, had nearly taken his life by frequent hæmorrhages. Astringents would control the hæmorrhage only for a short time. The actual cautery destroyed the tumor, but it was reproduced in one or two days. A piece of gutta percha was fitted to the roof of the mouth, and brought between the teeth, in such a manner, that when the lower jaw was shut upon the gutta percha, pressure would be made upon the tumor, and, also, there would be space sufficient between the teeth to supply him with food. The jaws were then bound firmly together. At the end of several days, the mouth was opened and examined, and it was found that the tumor had disappeared. Heated needles are sometimes passed in various directions through the tumor. Vaccinnation may be performed upon the part, and the inflammation will at times effect a cure. A seton may be passed through the diseased structure, and left till sufficient inflammation is produced. Injecting the tumor with some irritating fluid, may be tried, but this is apt to be attended with sloughing. These means are best adapted to the first and second variety.

*By arresting the circulation in the tumor.* This is accomplished, not entirely, but nearly so, by ligating the artery supplying the part. This will not always effect a cure, but there are times when it may be almost the only thing that can be done.

*By the destruction, or removal of the diseased tissue.* The actual cautery, or caustic applications, may be employed to effect this, but, in general, the knife, or the ligature is the instrument employed. The knife may be used in cases where there is not a great supply of blood from numerous arteries, or where the tumor is of a small size. The surgeon must expect, and be prepared for a profuse hæmorrhage, for generally there will be many arteries that will require the ligature. If the tumor is large, or if the surgeon expects great hæmorrhage, he should resort to the ligature. The best method is to pass a double ligature through the base of the tumor, and tying the different ends, the base of the tumor may be divided into two parts, each surrounded by a ligature. If the tumor is large, it may be best to pass a second double ligature, at right angles to the first, tying it in the same manner. Should the tumor be situated where a scar would cause much deformity, and if the skin has not become involved in the disease, a ligature may be passed, by means of a needle, around the base underneath the skin, avoiding, in a great degree, the scar. Sometimes the tumor is so flat, that a ligature cannot easily be applied; then two needles may be passed through the base of the tumor, at right angles to each other, and suffered to remain with their ends projecting; a ligature may then be placed around these, and thus be held in its place. Some of these means will generally accomplish a cure; but, unfortunately, in this disease, the surgeon sometimes is obliged to yield to a master, and see his patient pass from his hands, notwithstanding that his utmost skill and care has been exerted.

